# More than doubling the clinical benefit of each hour of therapist time: a randomised controlled trial of internet cognitive therapy for social anxiety disorder

**DOI:** 10.1017/S0033291722002008

**Published:** 2023-08

**Authors:** David M. Clark, Jennifer Wild, Emma Warnock-Parkes, Richard Stott, Nick Grey, Graham Thew, Anke Ehlers

**Affiliations:** 1 Department of Experimental Psychology, University of Oxford, Oxford, UK; 2 Department of Psychology, Institute of Psychiatry, Psychology and Neuroscience, Kings College, London, UK; 3 Sussex Partnership NHS Foundation Trust, Worthing, West Sussex, UK

**Keywords:** Cognitive behaviour therapy, IAPT, internet therapy, social anxiety

## Abstract

**Background:**

Cognitive therapy for social anxiety disorder (CT-SAD) is recommended by NICE (2013) as a first-line intervention. Take up in routine services is limited by the need for up to 14 ninety-min face-to-face sessions, some of which are out of the office. An internet-based version of the treatment (iCT-SAD) with remote therapist support may achieve similar outcomes with less therapist time.

**Methods:**

102 patients with social anxiety disorder were randomised to iCT-SAD, CT-SAD, or waitlist (WAIT) control, each for 14 weeks. WAIT patients were randomised to the treatments after wait. Assessments were at pre-treatment/wait, midtreatment/wait, posttreatment/wait, and follow-ups 3 & 12 months after treatment. The pre-registered (ISRCTN 95 458 747) primary outcome was the social anxiety disorder composite, which combines 6 independent assessor and patient self-report scales of social anxiety. Secondary outcomes included disability, general anxiety, depression and a behaviour test.

**Results:**

CT-SAD and iCT-SAD were both superior to WAIT on all measures. iCT-SAD did not differ from CT-SAD on the primary outcome at post-treatment or follow-up. Total therapist time in iCT-SAD was 6.45 h. CT-SAD required 15.8 h for the same reduction in social anxiety. Mediation analysis indicated that change in process variables specified in cognitive models accounted for 60% of the improvements associated with either treatment. Unlike the primary outcome, there was a significant but small difference in favour of CT-SAD on the behaviour test.

**Conclusions:**

When compared to conventional face-to-face therapy, iCT-SAD can more than double the amount of symptom change associated with each therapist hour.

Social anxiety disorder (SAD) is a disabling condition that can markedly interfere with work and social life (NICE, 2013). In the absence of treatment, it is one of the most persistent common mental health problems (Bruce et al., 2005). Randomised controlled trials in the UK, Europe and Japan have established that individual cognitive therapy for social anxiety disorder (CT-SAD) based on the Clark and Wells model (1995) is an effective treatment that compares favourably with several other active interventions. In particular, CT-SAD has been shown to be superior to three versions of group cognitive-behaviour therapy (CBT) (Ingul, Aune, & Nordahl, 2013; Mörtberg, Clark, Sundin, & Åberg Wistedt, 2007; Stangier, Heidenreich, Peitz, Lauterbach, & Clark, 2003), exposure therapy (Clark et al., 2006), interpersonal psychotherapy (Stangier, Schramm, Heidenreich, Berger, & Clark, 2011), psychodynamic therapy (Leichsenring et al., 2013), paroxetine (Nordahl et al., 2016), fluoxetine (Clark et al., 2003), and medication-based treatment as usual (Mörtberg et al., 2007). Yoshinaga et al. (2016) also reported that CT-SAD is effective in patients who remain symptomatic following anti-depressant treatment.

NICE (2013) recommends individual CT-SAD as a first-line intervention, in preference to group CBT and other psychological interventions. However, take-up of the treatment in routine healthcare systems has been hampered by demanding aspects of the treatment format. 14 weekly sessions are required, which is substantially more than the average number (7.5 sessions) that patients receive in the large-scale English Improving Access to Psychological Therapies (IAPT) treatment service (NHS Digital, 2021) or in similar services in Norway (Knapstad, Lervik, Sæther, Aarø, & Smith, 2020) and Australia (Baigent et al., 2020). Sessions are usually 90 min, rather than the standard 50–60 min, and therapists are expected to leave the office to accompany patients on some behavioural experiments. These are challenging requirements in busy routine services.

Internet-assisted therapy could potentially provide a solution to the challenges. A substantial literature shows that internet-based versions of CBT can be effective in a range of clinical conditions, including SAD (Andersson, 2016; Andersson, Carlbring, Titov, & Lindefors, 2019; Andrews et al., 2018). In internet-based therapy, the key skills in CBT are presented in learning modules that patients can access from home 24 h a day. Most programmes also include regular contact with a therapist. However, the contact is typically substantially briefer than in standard treatment and is remote (via in programme messaging, SMS texts, and/or by phone/ video link). Therapists do not accompany patients in out-of-office assignments. Instead within programme videos are used to illustrate, and motivate patients to complete such assignments.

Encouraged by the general success of internet-based CBT, we decided to develop an internet programme (iCT-SAD) that faithfully implements all the key procedures in CT-SAD. Although some of the existing internet CBT programmes for SAD (such as Andersson et al., 2006) present patients with the Clark and Wells (1995) model on which CT-SAD is based, none implement the full set of CT-SAD procedures, including video feedback and work on social trauma-related memories. In a small pilot study (Stott et al., 2013) improvements in social anxiety with the new iCT-SAD programme were broadly in line with those observed in previous trials of CT-SAD. We now report a randomised controlled trial that compares iCT-SAD with CT-SAD and a waitlist control group. We predicted iCT-SAD would be associated with similar improvements to those observed with CT-SAD but would require substantially less therapist time.

Two RCTs (Andrews, Davies, & Titov, 2011; Hedman et al., 2011) have compared other internet programmes against face-to-face CBT in patients with SAD and a third (Botella et al., 2010) compared internet CBT with face-to-face CBT in students with a more circumscribed fear of public speaking. Encouragingly, none of the trials found a difference in outcome between the internet and face-to-face therapy. However, the trials have some limitations that may have impacted on their ability to robustly test for differences. In the two SAD trials, the face-to-face treatment was group CBT, which is considered less cost-effective than individual CT-SAD (Mavranezouli et al., 2015). Neither trial included a behaviour test to assess anxiety in a live social interaction. All three trials failed to assess the competence with which the face-to-face treatment was delivered, although Hedman et al. (2011) showed good adherence to the treatment protocol. Therapeutic alliance was not assessed, and different therapists delivered the internet and face-to-face therapies. We address each of these limitations by comparing internet and standard face-to-face delivery of individual CT-SAD with the same therapists; including a behaviour test; assessing the competence with which the face-to-face therapy is delivered; and assessing the quality of the therapeutic alliances.

## Method

### Design

Patients were randomised to internet cognitive therapy for social anxiety disorder (iCT-SAD), standard cognitive therapy (CT-SAD), or waitlist control (WAIT) with continued GP care, each for 14 weeks. Patients who received either treatment were followed up for 12 months. Patients in the WAIT condition were randomised to iCT-SAD or CT-SAD at the end of the wait period if they still met the entry criteria for the trial (registration: ISRCTN95458747).

### Participants

Inclusion criteria: meets DSM-IV (American Psychiatric Association, 1994) criteria for SAD; SAD is the main problem; aged 18–65 years; internet access from home; no psychotropic medication or on a stable dose for at least 2 months (without clinical improvement) and willing to keep dose constant. Exclusion criteria: unable to attend weekly sessions; previous CBT or exposure therapy for SAD; immediate suicide risk; current substance dependency; current or past psychosis; borderline personality disorder.

Improving Access to Psychological Therapy (IAPT) services in the Oxford region and London referred patients for possible inclusion. Referrers and patients were told that controlled trials had established standard CT-SAD is effective and the study was investigating whether it was also effective when delivered online. Local ethics committees approved the study. All referrals were assessed with the SAD module of the Anxiety Disorders Interview Schedule (ADIS: Brown, Nardo, & Barlow, 1994), with the screener modules of the Structured Clinical Interview for DSM-IV, Axis-I (SCID-1; First, Spitzer, Gibbon, & Williams, 1995) and the screener questionnaire for Axis-II disorders (SCID-II: First, Gibbon, Spitzer, Williams, & Benjamin, 1997). If the SCID-I or SCID-II screeners indicated another disorder might be present, the relevant SCID module was administered. Regardless of screening responses, all patients were also assessed with the avoidant personality disorder section of the SCID-II. Diagnostic interviews were conducted by clinical psychologists who had received extensive training in the ADIS and SCID.

**Fig. 1.**
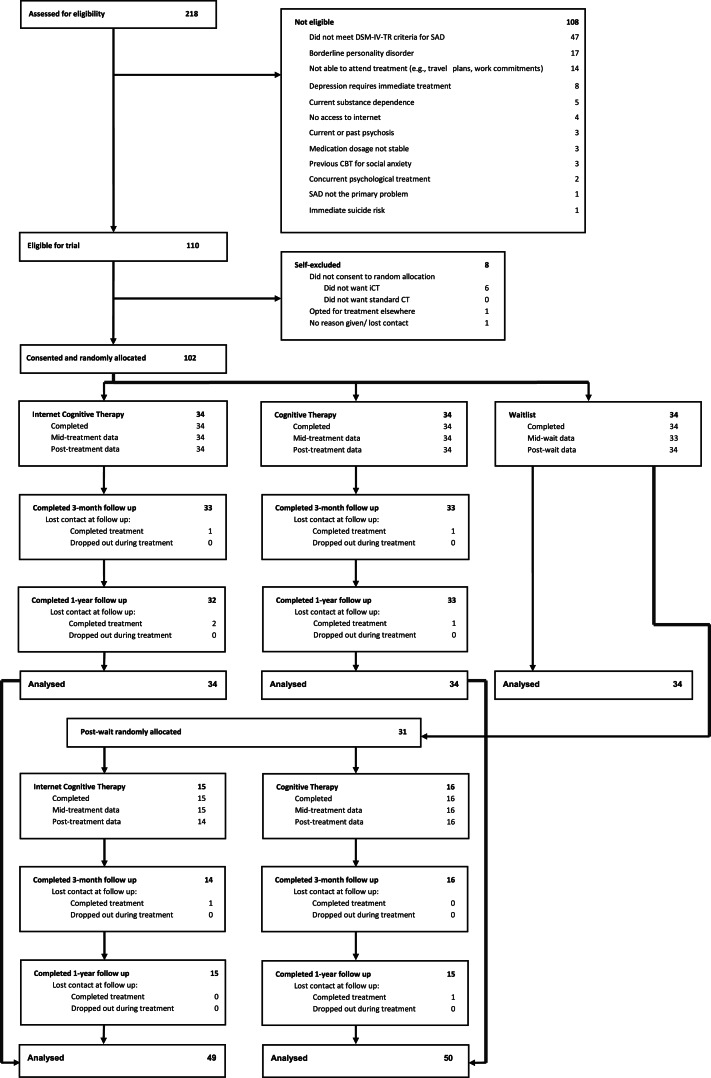
Flow chart of patients' progress through the trial.

Of 218 social anxiety patients referred between February 2013 and October 2014 for possible inclusion, 108 did not meet entry criteria. Figure 1 summarises the reasons. Eight patients who met the criteria declined to participate. After signing a consent form, the remaining 102 patients were allocated to one of the treatments or wait at a 1:1:1 ratio on a stratified random basis by an independent allocation office using the Minim computer programme. Stratification was by the severity of SAD (Liebowitz Social Anxiety Scale Score ⩽75 *v.* ⩾ 76; Liebowitz, 1987). At the end of WAIT, patients who still met trial criteria were randomly allocated to iCT (*n* = 15) or CT (*n* = 16) using the same stratification.

### Treatments

CT-SAD was the same as in Clark et al. (2006). Several procedures (therapist manual & video illustrations available at www.oxcadatresources.com) were used to reverse maintaining factors identified in Clark and Wells (1995) model of social phobia. These include: (a) an individualised version of Clark and Wells (1995) model; (b) experiential exercises to demonstrate the adverse effects of self-focused attention and safety behaviours; (c) systematic training in externally focused attention; (d) video feedback for restructuring distorted self-imagery (see Warnock-Parkes et al., 2017); (e) surveys of other peoples' attitudes to issues (such as blushing) that concern patients; (f) behavioural experiments in which patients test pre-specified negative predictions while dropping their safety behaviours and focusing externally; (g) decatastrophizing exercises; and (h) techniques (discrimination training and memory rescripting) for reducing the impact of early socially traumatic memories (see Wild and Clark, 2011). The protocol allowed up to 14 weekly (90 min) face-to-face therapy sessions and 3 booster sessions in the first 3 months of follow-up. Patients attended 12.8 (s.d. = 2.1) weekly treatment sessions (18.4 therapist hours) and 2.3 (s.d. = 0.9) booster sessions (2.8 therapist hours). Therapists frequently conducted behavioural experiments in therapy sessions, some of which were outside of the office. Total number of within-session behavioural experiments was 13.5 (s.d. = 6.1)

In iCT-SAD all the CT-SAD procedures were delivered within an internet programme (see Stott et al., 2013 for details). The programme comprises 8 core modules (online Supplementary Table S1) that patients complete in the first two weeks. Thereafter treatment is personalised with 16 additional modules (online Supplementary Table S1) on specific fearful beliefs or problems being available, depending on patients' concerns. iCT-SAD includes secure video conferencing with recording functionality to support conducting the self-focused attention and safety behaviour experiment and video feedback, as well as practising giving presentations to a virtual audience. Within module video clips illustrate how to set up and conduct behavioural experiments for particular fearful concerns. Patients were encouraged to do several behavioural experiments each week. Therapists scheduled short weekly phone calls with patients to review progress, assign new modules, deepen learning, and plan behavioural experiments. Summaries of calls were sent via the iCT-SAD secure messaging system, which was also used to provide encouragement and suggestions as appropriate. Reminders for behavioural experiments and questionnaire completion could be sent by within programme SMS. The core modules were released to all patients. On average 8.6 (range 0 to 12) additional modules were released. 81% of released modules were completed. Patients logged into the programme for a total of 43.9 h (s.d. = 24.2) and recorded 21.7 (s.d. = 18.6) completed behavioural experiments. Prior to the 14-week assessment, therapists had a mean of 12.4 (s.d. = 3.2) short weekly phone calls with their patients (2.8 h in total) and a mean of 2.5 (s.d. = 1.4) video calls (1.3 h in total), giving a total of 4.1 h of live therapist-patient contact. They sent an average of 38.2 (s.d. = 22.1) secure messages and 10.7 (s.d. = 12.2) SMS texts. Total estimated time spent supporting patients was 6.8 h up to week 14, with an additional 1 h during the booster period. There were no drop-outs (defined as disengaging before the midpoint) in either treatment.

### Therapists, supervision and treatment integrity

Three clinical psychologists (JW, EWP, RS) with substantial prior experience of CT-SAD and at least three practice cases with iCT-SAD each treated a third of the patients in each treatment group. DMC provided regular supervision. To check CT-SAD was consistently delivered to a high standard, a randomly selected session videotape from the treatment of each patient allocated to CT-SAD was rated by an independent expert (Louise Waddington) using the 15-item cognitive therapy competence rating scale for social phobia (CTCS-SP: Ginzburg *et al*., 2012; von Consbruch, Clark, & Stangier, 2012). Mean item score was 4.92 (s.d. = 0.47). No session was rated below 4 (on a 0–6 scale). All tapes were therefore rated ‘good’ to ‘excellent’. Competence in delivering iCT-SAD was not assessed as no appropriate measure was available.

At the start of the trial, 67 patients (66%) were medication free. The remaining 35 patients had been on a stable dose for at least two months without improvement and were asked not to change their medication. The medications were SSRIs (22 patients), beta-blockers (13 patients), benzodiazepines (2 patients) and another anti-depressant (1 patient). At the post-treatment/wait assessment, 24 of these patients were on the same medication and 11 had discontinued. 2 patients (1 iCT-SAD, 1 wait) started medication.

### Primary outcome measure

Online Supplementary Table S2 shows the assessment schedule. The pre-registered primary outcome measure was the social anxiety disorder composite. This was created by combining scores from six independent assessor and patient self-report scales of social anxiety, using Rosenthal and Rosnow's (1991) procedure, as in previous SAD trials (Clark et al., 2003, 2006). Patients' scores on each scale were standardised (*M* = 0, s.d. = 1) across pre-treatment and post-treatment assessments by converting to Z scores. The composite of each set on that occasion was the mean of the Z scores on that occasion. The individual scales that made up the composite are as follows. Independent assessors, who were blind to treatment allocation on each occasion, rated patients' fear and avoidance across a range of social situations using the ADIS for DSM-IV (Brown et al., 1994). Patients completed five standardised self-report SAD scales, which were: Liebowitz Social Anxiety Scale (LSAS: Baker, Heinrichs, Kim, & Hofmann, 2002), Social Phobia Inventory (SPIN: Connor et al., 2000), Social Phobia Scale (SPS: Mattick and Clarke, 1998), Social Interaction Anxiety Scale (SIAS: Mattick & Clarke, 1998), Fear of Negative Evaluation (FNE: Watson & Friend, 1969).

### Secondary outcome measures

#### Social anxiety disorder

Secondary outcomes included the proportion of patients who were rated by the independent assessor as no longer meeting diagnostic criteria for SAD and, for comparison with other studies, several commonly used responder and recovery criteria based on self-report scales. Participation in social activities and satisfaction with relationships was assessed with Alden and Taylor's (2011) scales.

#### Behaviour test

Patients also completed Heimberg's (2002) standardised behaviour test which comprises a conversation with two other people, followed by giving a speech to the same people. Patients rated their anxiety (0–100) during the tasks and afterwards rated how well (0–8) they thought they appeared using a 14-item checklist.

#### Anxious mood and depressed mood

The Generalized Anxiety Disorder scale (GAD-7: Spitzer, Kroenke, Williams, & Löwe, 2006) and the Patient Health Questionnaire (PHQ-9: Kroenke, Spitzer, & Williams, 2001) were used to assess anxious and depressed mood, respectively.

#### Functional impairment/interference

The Work and Social Adjustment Scale (WSAS: Mundt, Marks, Shear, & Greist, 2002) was used to assess interference with work, social, leisure and home life.

### Process measures

Four psychological processes targeted in treatment were assessed using the Social Cognitions Questionnaire (SCQ: frequency and belief in negative thoughts), Social Behaviours Questionnaire (SBQ: safety behaviours), Social Attitudes Questionnaire (SAQ: negative social anxiety-related assumptions) and Social Phobia Weekly Summary Scale (SPWSS: self-focused attention items). See Clark (2005) for details. These questionnaires are available at https://oxcadatresources.com/

### Non-specific therapy factors

At the end of the second week, therapist and patient independently completed a measure of therapeutic alliance (Agnew-Davies, Stiles, Hardy, Barkham, & Shapiro, 1998). Patients also completed Borkovec and Nau's (1972) treatment credibility and expectancy of improvement scales.

### Data analysis

Power calculations based on our previous trial of CT-SAD *v.* wait (Clark et al., 2006) indicated that a sample size of 34 per group would give power of 99% to detect differences between each treatment and wait. Comparisons between CT and iCT were based on all treated patients (including post-wait patients), with 50 patients per treatment giving 82% power to detect a clinically meaningful difference of 0.45 on the primary outcome measure. We analysed each continuous outcome with a linear mixed-effects regression model to account for repeated measures over time and include all available data from participants in the relevant randomisation. This method has the advantage of implicitly accounting for data missing at random. The models included categorical fixed factors of time (mid- intervention, post-intervention and, when relevant, 3 month and 12 month follow-up), and treatment condition (CT, iCT, Wait). The time-by-condition interaction was modelled as a fixed effect to allow estimation of treatment effect at each timepoint. Covariates were baseline score on the measure being analysed, and the PHQ. The latter was included as, by chance, there was a small imbalance in baseline depression between the groups^†^^1^. Participant was specified as a random effect to account for between-person variation. When analysing secondary outcome measures, the baseline score of the primary outcome measure was included as an additional covariate. The CT and iCT conditions were compared using the larger samples generated following the re-randomisation and treatment of the waitlist group participants after the wait period. These models included the data from the three-month and twelve-month follow-up timepoints. All models used restricted maximum likelihood estimation. Q-Q plots indicated that the normality of residuals assumption was met for all models. Between-group effect sizes (Cohen's *d*) were calculated by dividing the adjusted group difference by the pooled standard deviation.

To examine the potential mediating effect of process variables on clinical outcomes, we applied the analytic procedure described in Freeman et al. (2017), which follows the Baron and Kenny (1986) approach but uses linear mixed-effects models at each step to account for repeated measures within participants. Mid-treatment/wait scores on the composite process variable were examined as a candidate mediator of the relationship between randomisation (each treatment *v.* wait) and posttreatment/wait scores on the social anxiety composite. All models included baseline scores on the outcome, mediator, and PHQ as covariates, used the intention-to-treat sample and an alpha level of *p* = 0.05. Analyses were conducted in R version 3.5.1 (R Core Team, 2017) using the package ‘nlme’ (Pinheiro, Bates, DebRoy, & Sarkar, 2018).

Linear regression identified possible predictors of outcome within each treatment. Post-treatment scores on the primary outcome measure were related to each putative predictor while controlling for pre-treatment scores.

## Results

### Characteristics of patients

Patients' mean age was 32.2 (s.d. = 8.3) and the mean duration of SAD was 18.4 years (s.d. = 9.6). Fifty-two per cent were female. Fifty-four per cent were married, cohabiting or in a long-term relationship. Eighty-eight per cent were Caucasian. Seventy-six per cent were employed, 14% were students and 10% were unemployed. Fourteen per cent left school by age 16, 21% completed high school and 65% had some higher education. Fifty-five per cent met diagnostic criteria for one or more additional current axis-I disorders. The main co-morbid axis-I disorders were: depressive disorder (30%), generalised anxiety disorder (20%), somatoform disorder (8%), specific phobia (8%), panic disorder (6%), alcohol or substance abuse (4%), agoraphobia (2%), obsessive-compulsive disorder (2%), PTSD (1%) and anorexia (1%). Eighty-one per cent met the criteria for one or more personality disorders, which were avoidant (73%), obsessive (30%), and paranoid (13%). There were no significant differences between the groups on these characteristics.

### Treatment credibility and therapeutic alliance

Patient ratings of treatment credibility (item means CT-SAD 8.0, s.d. = 1.1; iCT-SAD 7.8 s.d. = 1.2) and therapeutic alliance (CT-SAD 73.1, s.d. = 8.3; iCT-SAD 74.1, s.d. = 7.6) were high and did not differ. However, therapists rated the therapeutic alliance (CT-SAD 77.1, s.d. = 7.0; iCT-SAD 71.3, s.d. = 7.3. *t*_(65)_ = 3.3, *p* < 0.001) more highly in CT-SAD.

### Comparisons between treatments and waitlist control group

Table 1 shows relevant scores at each assessment. On the primary outcome measure (SAD composite) both treatments were superior to the waitlist, with large effect sizes (*d* = 2.20 for iCT-SAD and 2.38 for CT-SAD) at post-treatment/wait. Similar results were observed for the behaviour test (Table 2), the other secondary outcome measures (online Supplementary Table S3) and the process composite (Table 1).

**Table 1.** Comparisons between the treatments and the wait list control group on the primary outcome and the process composite

Measure	Time	Unadjusted mean (s.d.) (*N*)			Adjusted difference (95% CI), *p* value, *d*	
		iCT	CT	Wait	iCT *v.* Wait	CT *v.* Wait
Social anxiety composite	Pre	0.82 (0.58) (34)	0.71 (0.89) (34)	0.69 (0.67) (34)		
	Mid	−0.35 (0.77) (34)	−0.80 (1.17) (34)	0.77 (0.90) (33)	−1.22 (−1.61 to −0.83), <0.001, 1.44	−1.57 (−1.96 to −1.18), <0.001, 1.48
	Post	−1.24 (0.84) (34)	−1.71 (1.01) (34)	0.64 (0.92) (34)	−1.97 (−2.36 to −1.58), <0.001, 2.20	−2.33 (−2.72 to −1.94), <0.001, 2.38
Process composite	Pre	0.86 (0.58) (34)	0.74 (0.90) (34)	0.75 (0.71) (34)		
	Mid	−0.63 (0.87) (34)	−1.01 (0.87) (34)	0.77 (0.90) (33)	−1.48 (−1.86 to −1.10), <0.001, 1.65	−1.80 (−2.19 to −1.42), <0.001, 2.01
	Post	−1.33 (0.87) (34)	−1.74 (0.81) (34)	0.66 (0.97) (34)	−2.05 (−2.43 to −1.67), <0.001, 2.19	−2.41 (−2.79 to −2.03), <0.001, 2.65

iCT, Internet-based Cognitive Therapy; CT, Standard (face-to-face) Cognitive Therapy.

Adjusted mean differences based on linear mixed-effects models adjusted for baseline scores. *d* is the standardised effect size (Cohen's d), calculated using the pooled standard deviation.

**Table 2.** Behaviour Test: comparisons between the treatments and wait-list control condition

Measure	Time	Unadjusted mean (s.d.) (*N*)			Adjusted difference (95% CI), *p* value, *d*	
		iCT	CT	Wait	iCT *v.* Wait	CT *v.* Wait
Anxiety ratings	Pre	64.19 (14.94) (34)	63.21 (17.51) (34)	65.95 (19.22) (34)		
	Post	32.37 (15.71) (32)	21.85 (16.21) (34)	58.82 (19.66) (32)	26.87 (19.96–33.78), <0.001, 1.49	36.03 (29.14–42.92), <0.001, 1.97
Behaviour checklist	Pre	3.32 (1.18) (34)	3.37 (1.10) (34)	3.12 (1.34) (34)		
	Post	5.49 (0.98) (32)	5.91 (1.06) (34)	3.57 (1.12) (32)	−1.88 (−2.29 to −1.46), <0.001, 1.75	−2.25 (−2.66 to −1.84), <0.001, 2.04

iCT, Internet-based Cognitive Therapy; CT, Standard (face-to-face) Cognitive Therapy.

Adjusted mean differences based on linear mixed-effects models adjusted for baseline scores. *d* is the standardised effect size (Cohen's *d*), calculated using the pooled standard deviation. For the behaviour checklist a higher score indicates better performance. Anxiety and behaviour checklist scores are averaged across the conversation and the speech. For the conversation, patients are told they will be meeting two people and should talk with them as though they met them at a party. For the speech the patient stands while the two audience members sit. Patients are asked to talk about a topic that they decide in advance; either some current event, or hobbies, or something work-related. For both the conversation and the speech, patients are given 2 min to prepare before entering the room.

*Deterioration rates* were calculated using IAPT criteria (NCCMH, 2021, p. 40). No patients in either treatment deteriorated between pre and post, but 9% (*n* = 3) in the wait group deteriorated (1 due to a reliable increase on the SPIN and 2 due to a reliable increase on the PHQ).

### Comparisons between the two treatments

Table 3 shows the scores at each assessment for the primary outcome, the continuous secondary outcome measures, and the process composite. (see online Supplementary Table S4 for components of the composites). On the primary outcome, there were no significant differences between internet (iCT-SAD) and standard (CT-SAD) cognitive therapy at any time point (*d*s range from 0.22 to 0.31). There were also no significant differences on the process composite at any timepoint (*d*s range from 0.19 to 0.38). In contrast to the lack of differences on the primary social anxiety measure, there as a significant difference in favour of CT-SAD on one of the two measures in the post-treatment behaviour test (self-reported anxiety, *p* = 0.011, *d* = 0.47). CT-SAD was also associated with a significantly lower depression score at post-treatment (*p* = 0.002, *d* = 0.64) but not at the 3mth or 12mth follow-ups. There were no significant differences between the treatments on any other measure at post-treatment or follow-up.

**Table 3.** Comparisons between internet (iCT) and standard face-to-face treatment (CT) on primary outcome, process composite and secondary outcomes

Measure	Time	Unadjusted mean (s.d.) (*N*)		Adjusted difference (95% CI), *p* value, *d*
		iCT	CT	
Social anxiety composite	Pre	0.80 (0.66) (49)	0.71 (0.85) (50)	
	Mid	−0.35 (0.87) (49)	−0.68 (1.11) (50)	−0.28 (−0.65 to 0.09), 0.142, 0.22
	Post	−1.27 (0.94) (48)	−1.60 (0.99) (50)	−0.30 (−0.67 to 0.07), 0.112, 0.31
	3 m	−1.53 (0.88) (47)	−1.75 (1.01) (49)	−0.23 (−0.61 to 0.14), 0.216, 0.24
	12 m	−1.41 (0.94) (48)	−1.73 (1.01) (48)	−0.31 (−0.68 to 0.07), 0.109, 0.31
Process composite	Pre	0.87 (0.67) (49)	0.70 (0.87) (50)	
	Mid	−0.59 (0.94) (49)	−0.88 (0.88) (50)	−0.25 (−0.60 to 0.10), 0.158, 0.27
	Post	−1.32 (0.88) (48)	−1.65 (0.76) (50)	−0.31 (−0.67 to 0.04), 0.079, 0.38
	3 m	−1.60 (0.75) (47)	−1.76 (0.81) (49)	−0.17 (−0.53 to 0.18), 0.334, 0.22
	12 m	−1.39 (0.98) (48)	−1.59 (0.98) (48)	−0.19 (−0.55 to 0.16), 0.282, 0.19
Social participation	Pre	40.94 (12.52) (49)	43.01 (13.56) [50)	
	Mid	53.73 (13.36) (48)	54.17 (15.22) (48)	−0.13 (−5.33 to 5.08), 0.962, 0.01
	Post	60.93 (12.90) (46)	60.39 (15.02) (50)	−0.81 (−6.01 to 4.39), 0.758, 0.06
	3 m	60.93 (11.79) (46)	61.02 (13.68) (49)	−0.22 (−5.44 to 5.00), 0.934, 0.02
	12 m	60.75 (15.90) (48)	63.91 (16.55) (48)	2.44 (−2.76 to 7.65), 0.354, 0.15
Social satisfaction	Pre	17.59 (5.30) (49)	18.74 (5.73) (50)	
	Mid	21.10 (5.56) (48)	21.96 (5.21) (48)	0.28 (−1.67 to 2.35), 0.786, 0.05
	Post	23.54 (5.46) (46)	24.75 (5.39) (50)	0.69 (−1.26 to 2.75), 0.502, 0.13
	3 m	23.52 (5.21) (46)	25.02 (5.76) (49)	0.90 (−1.05 to 2.96), 0.383, 0.16
	12 m	23.23 (6.30) (48)	25.19 (6.70) (48)	1.43 (−0.51 to 3.50), 0.164, 0.22
WSAS	Pre	3.45 (1.45) (49)	2.92 (1.05) (50)	
	Mid	2.47 (1.66) (49)	1.75 (1.01) (50)	−0.51 (−0.98 to −0.03), 0.036, 0.37
	Post	1.77 (1.62) (48)	1.14 (0.93) (50)	−0.44 (−0.92 to 0.03), 0.066, 0.33
	3 m	1.44 (1.15) (47)	1.15 (1.07) (49)	−0.16 (−0.63 to 0.32), 0.517, 0.14
	12 m	1.43 (1.32) (48)	1.04 (1.15) (48)	−0.20 (−0.68 to 0.28), 0.405, 0.16
GAD-7	Pre	9.82 (5.32) (49)	8.72 (5.06) (50)	
	Mid	5.61 (4.41) (49)	3.76 (3.35) (50)	−1.62 (−2.90 to −0.35), 0.013, 0.41
	Post	3.75 (3.81) (48)	2.48 (2.53) (50)	−1.06 (−2.34 to 0.22), 0.104, 0.33
	3 m	2.74 (3.01) (47)	2.88 (3.46) (49)	0.33 (−0.96to 1.63), 0.608, 0.10
	12 m	3.46 (3.28) (48)	2.41 (2.84) (48)	−0.81 (−2.10 to 0.48), 0.214, 0.26
PHQ-9	Pre	9.12 (5.47) (49)	7.27 (5.38) (50)	
	Mid	5.82 (4.64) (49)	3.00 (2.84) (50)	−2.54 (−3.83 to −1.24), <0.001, 0.65
	Post	4.02 (3.95) (48)	1.68 (2.32) (50)	−2.08 (−3.38 to −0.78), 0.002, 0.64
	3 m	3.15 (3.01) (47)	2.10 (2.54) (49)	−0.86 (−2.16 to 0.45), 0.198, 0.30
	12 m	3.21 (3.46) (48)	2.35 (3.05) (48)	−0.57 (−1.88 to 0.74), 0.392, 0.17
Behaviour test – anxiety ratings	Pre	62.60 (17.10) (49)	62.48 (17.13) (50)	
	Post	31.55 (16.89) (46)	22.74 (17.24) (49)	−8.11 (−14.31 to −1.91), 0.011, 0.47
Behaviour test – behaviour checklist	Pre	3.35 (1.17) (49)	3.45 (1.08) (50)	
	Post	5.36 (1.04) (46)	5.85 (1.10) (49)	0.39 (−0.01 to 0.79), 0.055, 0.36

Notes. iCT = Internet-based Cognitive Therapy, CT = Standard (face-to-face) Cognitive Therapy. Table includes everyone who was randomized to iCT or CT, either immediately or at the end of the waitlist. WSAS = Work and Social Adjustment Scale, GAD-7 = Generalised Anxiety Disorder Questionnaire, PHQ-9 = Patient Health Questionnaire. Adjusted mean differences based on linear mixed effects models adjusted for baseline scores. *d* is the standardised effect size (Cohen’s d), calculated using the pooled standard deviation.

Table 4 shows the proportions of patients who lost the diagnosis of SAD and that met the IAPT criteria for recovery (NCCMH, 2021) at post-treatment and follow-up. There were no significant differences between the treatments at either assessment. The loss of diagnosis rates (72% to 91%) are higher than the recovery rates based on IAPT criteria (59% to 82%) as the latter require individuals to drop below the clinical cut-off on both social anxiety and depression and also code patients with missing data as not recovered (NCCMH, 2021, p. 39). online Supplementary Table S5 shows the proportions of individuals meeting other responder and remission criteria that are commonly cited in the literature.

**Table 4.** Recovery rates (loss of diagnosis and IAPT criteria)

Assessment	iCT		CT	
	%	(*n*)^a^	%	(*n*)^a^
Loss of SAD diagnosis				
Pretreatment	0	(0/49)	0	(0/50)
Posttreatment	72	(33/46)	84	(42/50)
3 month Follow-up	84	(37/44)	91	(42/46)
12 month Follow-up	84	(34/44)	87	(39/45)
IAPT Recovery Criteria^b^				
Pretreatment	0	(0/49)	0	(0/50)
Posttreatment	59	(29/46)	72	(36/50)
3 month Follow-up	65	(32/47)	80	(40/49)
12 month Follow-up	67	(33/48)	75	(36/48)

a Numerator is the number of people meeting the relevant criteria. Denominator is the number of people with data on the relevant measure.

b IAPT recovery requires patients to initially score above the clinical cut-off on the Social Phobia Inventory (19) and/or PHQ (10) and to subsequently score below the clinical cut-off on *both* the SPIN and the PHQ. All patients scored above the clinical threshold on SPIN at pretreatment and hence are classified as clinical cases at that time point. In line with the IAPT manual (National Collaborating Centre for Mental Health, 2021), patients who did not have a SPIN or PHQ score available at post-treatment or follow-up are assumed to have not recovered. So, the denominators for the % recovery values are *n* = 49 for iCT and *n* = 50 for CT.

Most patients (72%, *n* = 71/99) met the diagnostic criteria for avoidant personality disorder at pre-treatment. For most (60/71), the SCID-II diagnostic interview was repeated at one-year follow-up. At that point, 82% (49/60) had lost the diagnosis of avoidant PD.

To further explore the apparent lack of difference between the two treatments on the primary outcome measure, we adopted Rogers, Howard, and Vessey's (1993) confidence interval approach to equivalence testing. Setting alpha at 0.05, we were able to reject the hypothesis that iCT-SAD could be more than 25% less effective than CT-SAD at post-treatment or follow-up.

### Therapeutic change per hour of therapy

**Fig. 2.**
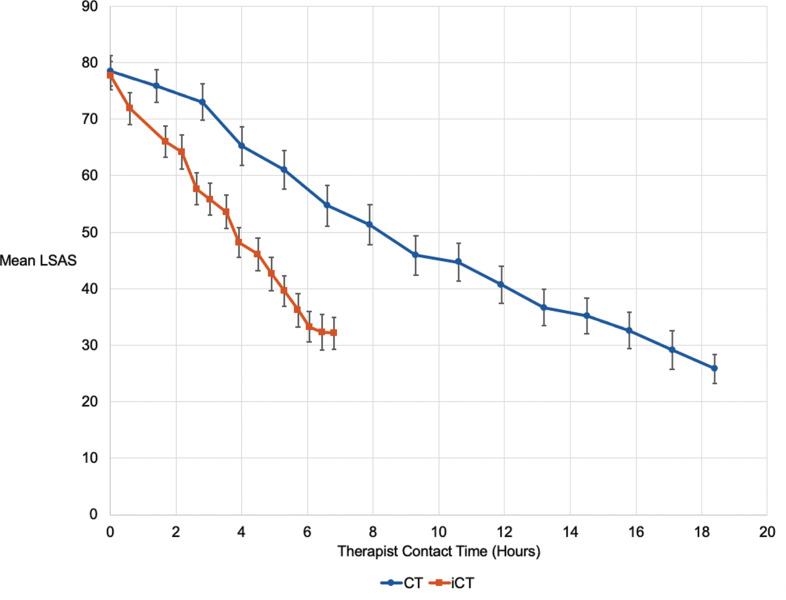
Means and standard errors for weekly Liebowitz Social Anxiety Scale (LSAS) plotted against cumulating therapist time.

Patients completed the Liebowitz Social Anxiety Scale (LSAS) each week during treatment. Figure 2 shows the weekly scores plotted against cumulating therapist time. By the end of treatment, iCT patients had dropped 45.5 points on the LSAS after an average of 6.45 h contact with their therapist. In CT, 15.8 h of therapist contact were required to achieve the same drop on the LSAS. iCT is therefore associated with 2.45 times more symptom change per hour of therapist contact time.

### Mediation analysis

To examine whether the effectiveness of the two treatments was partly due to their ability to change processes specified in the Clark and Wells (1995) cognitive model, we conducted mediation analyses. For both iCT-SAD and CT-SAD scores on the process composite at mid-treatment/wait mediated the relationship between randomisation (treatment *v.* wait) and scores on the primary outcome measure at post-treatment/wait (see Table 5). It, therefore, appears that each treatment partly works by changing the process variables.

**Table 5.** Mediation analysis

	Total effect		Direct effect		Indirect effect		% Mediated
	Adjusted difference (s.e.) (95% CI)	*p*	Adjusted difference (s.e.) (95% CI)	*p*	Adjusted difference (s.e.) (95% CI)	*p*	
Mediator = Process Composite at Mid; Outcome = Social Anxiety Composite at Post							
iCT *v.* Wait	1.97 (0.20) (1.58–2.36)	<0.001	0.79 (0.16) (0.48–1.10)	<0.001	−1.17 (0.17) (−1.51 to −0.83)	<0.001	59
CT *v.* Wait	2.33 (0.20) (1.94–2.73)	<0.001	0.92 (0.17) (0.59–1.26)	<0.001	−1.41 (0.18) (−1.77 to −1.05)	<0.001	60

*Notes*. *N* = 34 per condition. Coefficients estimated using linear mixed-effects models, with total, direct, and indirect effects calculated following Baron and Kenny (1986). Models included baseline scores on the mediator and outcome variables as fixed covariates, and a random effect of participant.

### Predictors of treatment response

Patients who rated their treatment as more credible and thought they had a better therapeutic relationship with their therapist at week 2 scored lower on the social anxiety composite at post-treatment in both iCT (credibility: standardised *β* = −0.373, *p* = 0.01; therapeutic alliance: standardised *β* = −0.0470, *p* = 0.001) and CT (credibility: standardised beta = −0.342, *p* = 0.01; therapeutic alliance: standardised beta = −0.459, *p* = 0.001). Early therapist ratings of the therapeutic relationship did not predict outcome in CT (standardised *β* = −0.175, *p* = 0.225) but did predict outcome in iCT (standardised *β* = 0.349, *p* = 0.015). Within iCT, individuals who practised giving presentations to a virtual audience more frequently had better outcomes (standardised *β* = −0.420, *p* = 0.003). The presence of avoidant personality disorder did not predict outcome in either treatment.

## Discussion

The primary aim of the trial was to determine whether iCT-SAD can achieve similar outcomes to well-conducted individual CT-SAD in less therapist time. Competence ratings for CT-SAD confirmed it was delivered to a high standard and the observed improvements were comparable to those in our previous trial (Clark et al., 2006). iCT-SAD was also associated with large improvements and did not differ significantly from CT-SAD on the primary outcome, despite not requiring the delivery features of CT-SAD that services find challenging, such as 90 min sessions and accompanying patients on behavioural experiments outside the office. Week by week tracking of social anxiety indicated that the full symptom change associated with iCT-SAD was achieved after only 6.45 h of therapist time whereas CT-SAD required 15.8 h to achieve the same amount of improvement. It, therefore, appears that iCT-SAD achieves 2.45 times more symptom change per hour of therapist time. The total therapist time required for iCT-SAD is similar to the average in IAPT services. Removal of the need to accompany patients on out-of-the-office assignments is also likely to make it easier to use in routine healthcare settings.

Cognitive therapy aims to treat SAD by changing the key maintenance processes specified in Clark and Wells (1995) and similar (Hofmann, 2007; Rapee & Heimberg, 1997) cognitive models. To test whether improvements associated with the treatment are at least partly attributable to its ability to change these processes, a mediation analysis was conducted. For both internet and face-to-face versions, change in processes specified in the models accounted for around 60% improvement associated with treatment. As well as supporting the cognitive models on which treatment is based, this finding suggests that therapists should carefully track and maximise change in self-focused attention, negative social anxiety-related beliefs and safety behaviours during treatment. To facilitate this, the iCT-SAD programme automatically graphs change in these variables.

Research has shown that the quality of a therapeutic relationship plays an important role in the outcome of many psychological treatments (Norcross, 2011), including some (see Berger, 2017), but not all (Zalaznik *et al*., 2021), of those delivered over the internet. We also found a significant relationship between early ratings of therapeutic alliance and eventual outcome. It might be thought that the remote support used in iCT-SAD would have an adverse effect on the therapeutic relationship. However, patient ratings of the therapeutic alliance were as high in iCT-SAD as in CT-SAD. Therapist ratings were slightly lower for iCT-SAD, perhaps because the type of communication was something that they were less familiar with. Initial ratings of the credibility of psychological treatment have also often been shown to predict outcome (Devilly & Borkovec, 2000) and did so in iCT-SAD. Therapists should therefore ensure that when patients are allocated to iCT-SAD any concerns about its credibility are identified and addressed.

This trial included a behaviour test that provided additional insights. iCT-SAD was associated with large reductions in anxiety during the test (*d* = 1.47). In contrast to the other primary and secondary social anxiety measures, there was also a modest (*d* = 0.47), but significant, difference in favour of CT-SAD. In view of this finding, we suggest future evaluations of internet treatments for SAD include a behaviour test. However, the interpretation of the difference in favour of CT-SAD in this study is complicated by the fact that the test was conducted in the clinic where CT-SAD patients had their treatment. It is unclear whether the additional familiarity that CT-SAD patients had with the setting and staff contributed to the difference. To avoid this ambiguity, behaviour tests in future studies should be conducted elsewhere.

### Limitation and further research

To provide a rigorous test of internet delivery we used the same experienced therapists for internet and face-to-face therapy. Further studies are required to determine whether including all the key therapy procedures in the internet programme will enable therapists in routine services who have had less prior experience with standard cognitive therapy to achieve similarly positive results with iCT-SAD.

## Footnotes

† The notes appear after the main text.

## Notes

1 Not including baseline PHQ as a covariate produced the same results as reported here.

## Supplementary material

For supplementary material accompanying this paper visit https://doi.org/10.1017/S0033291722002008. click here to view supplementary material
