# Supplementary material for: More than doubling the clinical benefit of each hour of therapist time: a randomised controlled trial of internet cognitive therapy for social anxiety disorder
Source: Psychol Med. 2022 Jul 15;53(11):5022–32. doi: 10.1017/S0033291722002008 (PMC10476054; doi:10.1017/S0033291722002008)
Supplement: Supplementary file 1 [file S0033291722002008sup001.docx]

**Supplementary materials**

Table S1 **iCT-SAD Modules**

*_____________________________________________________________________________________________________*

*Core Modules (Allocated to all patients)*

Introducing the treatment

Getting started

Feeling self-conscious

Safety behaviours

Attention & safety behaviours experiment

Watching your conversation videos

Getting out of your head and into the world

Behavioural experiments

*Additional Modules for particular concerns and problems*

Blushing

Sweating

Shaking

Having conversations

Feeling boring

Feeling stupid

Feeling responsible for others enjoyment

De-catastrophising

Worrying in advance

Going over social situations after they’ve happened

Leaving the past behind

Managing my inner critic

Self-esteem

Managing my mood

Giving myself credit

My therapy blueprint

*Modules for to help patients prepare for follow-up sessions*

Preparing for first follow-up

Preparing for second follow-up

Preparing for third follow-up

_________________________________________________________________________________________________

| Table S2 Assessment Schedule | | | | | | | | | | | | | | | | | |
| --- | --- | --- | --- | --- | --- | --- | --- | --- | --- | --- | --- | --- | --- | --- | --- | --- | --- |
|  | Time | | | | | | | | | | | | | | | | |
|  | Pre |  |  | (Week) | |  |  | Mid |  |  | (Week) | |  |  | Post |  |  |
|  |  | 1 | 2 | 3 | 4 | 5 | 6 | 7 | 8 | 9 | 10 | 11 | 12 | 13 | 14 | 3mthFU | 12mthFU |
| Measure |  |  |  |  |  |  |  |  |  |  |  |  |  |  |  |  |  |
|  | | | | | | | | | | | | | | | | | |
| LSAS | **X** | **X** | **X** | **X** | **X** | **X** | **X** | **X** | **X** | **X** | **X** | **X** | **X** | **X** | **X** | **X** | **X** |
| SPIN | **X** |  |  |  |  |  |  | **X** |  |  |  |  |  |  | **X** | **X** | **X** |
| SIAS | **X** |  |  |  |  |  |  | **X** |  |  |  |  |  |  | **X** | **X** | **X** |
| SPS | **X** |  |  |  |  |  |  | **X** |  |  |  |  |  |  | **X** | **X** | **X** |
| FNE | **X** |  |  |  |  |  |  | **X** |  |  |  |  |  |  | **X** | **X** | **X** |
| ADIS-SAD | **X** |  |  |  |  |  |  |  |  |  |  |  |  |  | **X** | **X** | **X** |
| SCQ | **X** | X | X | X | X | X | X | **X** | X | X | X | X | X | X | **X** | **X** | **X** |
| SBQ | **X** |  |  |  |  |  |  | **X** |  |  |  |  |  |  | **X** | **X** | **X** |
| SAQ | **X** |  |  |  |  |  |  | **X** |  |  |  |  |  |  | **X** | **X** | **X** |
| SFA | **X** | X | X | X | X | X | X | **X** | X | X | X | X | X | X | **X** | **X** | **X** |
| Soc Sat | **X** |  |  |  |  |  |  | **X** |  |  |  |  |  |  | **X** | **X** | **X** |
| Soc Part | **X** |  |  |  |  |  |  | **X** |  |  |  |  |  |  | **X** | **X** | **X** |
| Behaviour Test | **X** |  |  |  |  |  |  |  |  |  |  |  |  |  | **X** |  |  |
| PHQ | **X** | X | X | X | X | X | X | **X** | X | X | X | X | X | X | **X** | **X** | **X** |
| GAD | **X** | X | X | X | X | X | X | **X** | X | X | X | X | X | X | **X** | **X** | **X** |
| Note. LSAS = Liebowitz Social Anxiety Scale, SPIN = Social Phobia Inventory, SIAS = Social Interaction Anxiety Scale, SPS = Social Phobia Scale, FNE = Fear of Negative Evaluation Scale, ADIS = Anxiety Disorders Interview Schedule (Social Anxiety Scale), SCQ = Social Cognitions Questionnaire, SBQ = Social Behaviours Questionnaire, SAQ = Social Attitudes Questionnaire, SFA = self-focused attention, SocSat = social satisfaction, SocPart = social participation, PHQ = patient health questionnaire, GAD = generalized anxiety disorder. The WAIT group completed the pre, mid, and post assessments. The two treatment groups completed all assessments. **X** = measure collected as part of the trial dataset. X= measure collected for clinical purposes to help guide the delivery of CT and iCT and/or as a national requirement for IAPT services. | | | | | | | | | | | | | | | | | |

Table S3. Comparison between the treatments and wait-list control condition on secondary outcome measures

| Measure | Time |  | Unadjusted Mean (SD) [N] | | |  | Adjusted difference [95%CI],  p value, *d* | |
| --- | --- | --- | --- | --- | --- | --- | --- | --- |
|  |  |  | iCT | CT | Wait |  | iCT vs Wait | CT vs Wait |
| Social Participation | Pre |  | 41.38  (13.48) [34] | 44.24  (15.03) [34] | 44.46  (13.86) [34] |  |  |  |
|  | Mid |  | 53.27  (13.24) [33] | 56.06  (16.05) [32] | 41.12  (12.74) [32] |  | -14.30 [-19.84, -8.76],  <.001, 1.08 | -15.45 [-21.05, -9.85],  <.001, 1.05 |
|  | Post |  | 60.84  (12.37) [32] | 63.39  (16.63) [34] | 40.49  (12.82) [34] |  | -21.84 [-27.34, -16.34],  <.001, 1.71 | -22.92 [-28.40, -17.44],  <.001, 1.52 |
|  |  |  |  |  |  |  |  |  |
| Social Satisfaction | Pre |  | 18.71  (5.07) [34] | 18.85  (5.88) [34] | 17.76  (5.84) [34] |  |  |  |
|  | Mid |  | 21.58  (5.61) [33] | 22.62  (5.38) [32] | 17.69  (6.48) [32] |  | -2.97 [-4.99, -0.95],  .012, 0.48 | -4.02 [-6.08, -1.96],  <.001, 0.66 |
|  | Post |  | 23.78  (5.09) [32] | 25.60  (5.47) [34] | 17.34  (5.87) [34] |  | -5.50 [-7.52, -3.48],  <.001, 0.98 | -7.47 [-9.48, -5.46],  <.001, 1.30 |
|  |  |  |  |  |  |  |  |  |
| WSAS | Pre |  | 3.31  (1.39) [34] | 2.90  (1.01) [34] | 3.23  (1.21) [34] |  |  |  |
|  | Mid |  | 2.45  (1.56) [34] | 1.57  (0.95) [34] | 3.29  (1.41) [33] |  | 0.87 [0.32, 1.42],  .006, 0.58 | 1.50 [0.94, 2.05],  <.001, 1.23 |
|  | Post |  | 1.79  (1.53) [34] | 0.96  (0.85) [34] | 3.25  (1.48) [34] |  | 1.51 [0.97, 2.06],  <.001, 0.99 | 2.10 [1.54, 2.65],  <.001, 1.71 |
|  |  |  |  |  |  |  |  |  |
|  |  |  |  |  |  |  |  |  |
|  |  |  |  |  |  |  |  |  |
| GAD-7 | Pre |  | 9.82  (5.28) [34] | 8.44  (5.37) [34] | 9.47  (5.22) [34] |  |  |  |
|  | Mid |  | 5.38  (3.85) [34] | 3.18  (2.43) [34] | 9.09  (5.78) [33] |  | 3.84 [2.12, 5.56],  <.001, 0.77 | 5.38 [3.64, 7.11],  <.001, 1.20 |
|  | Post |  | 3.76  (3.31) [34] | 2.18  (2.26) [34] | 9.44  (5.24) [34] |  | 5.86 [4.15, 7.57],  <.001, 1.32 | 6.78 [5.06, 8.50],  <.001, 1.66 |
|  |  |  |  |  |  |  |  |  |
| PHQ-9 | Pre |  | 9.21  (5.75) [34] | 6.82  (5.10) [34] | 8.43  (5.04) [34] |  |  |  |
|  | Mid |  | 5.53  (4.17) [34] | 2.47  (2.26) [34] | 8.76  (5.62) [33] |  | 3.46 [1.71, 5.21],  <.001, 0.69 | 5.75 [3.98, 7.52],  <.001, 1.33 |
|  | Post |  | 3.79  (3.20) [34] | 1.41  (1.56) [34] | 8.19  (5.40) [34] |  | 4.65 [2.90, 6.40],  <.001, 1.03 | 6.27 [4.51, 8.03],  <.001, 1.55 |
|  |  |  |  |  |  |  |  |  |

*Notes.* WSAS = Work and Social Adjustment Scale, GAD-7 = Generalised Anxiety Disorder Questionnaire, PHQ-9 = Patient Health Questionnaire, iCT = Internet-based Cognitive Therapy, CT = Standard (face-to-face) Cognitive Therapy. Adjusted mean differences based on linear mixed effects models adjusted for baseline scores. *d* is the standardised effect size (Cohen’s d), calculated using the pooled standard deviation.

Table S4. Comparisons between internet (iCT) and standard face-to-face treatment (CT) on the measures that make up the social anxiety composite (primary outcome measure) and the process composite.

| Measure | Time |  | Unadjusted Mean (SD) [N] | |  | Adjusted difference [95%CI], p value, *d* |
| --- | --- | --- | --- | --- | --- | --- |
|  |  |  | iCT | CT |  |  |
| *Components of social anxiety composite* | | | | | | |
| SPIN | Pre |  | 40.37 (8.83) [49] | 40.63 (10.57) [50] |  |  |
|  | Mid |  | 26.79 (9.52) [48] | 24.57 (12.97) [48] |  | -2.40 [-6.73, 1.93], .274, 0.21 |
|  | Post |  | 17.46 (9.77) [46] | 14.56 (11.46) [50] |  | -3.48 [-7.81, 0.85], .113, 0.32 |
|  | 3m |  | 14.64 (10.09) [47] | 12.53 (9.76) [49] |  | -2.53 [-6.86, 1.80], .248, 0.25 |
|  | 12m |  | 14.42 (10.43) [48] | 11.97 (10.61) [48] |  | -2.90 [-7.23, 1.43], .186, 0.27 |
| SIAS | Pre |  | 50.71 (11.56) [49] | 48.33 (14.04) [50] |  |  |
|  | Mid |  | 37.48 (13.57) [48] | 34.17 (14.21) [48] |  | -2.65 [-7.45, 2.15], .276, 0.19 |
|  | Post |  | 25.89 (12.85) [46] | 23.09 (12.92) [50] |  | -2.67 [-7.47, 2.13], .274, 0.21 |
|  | 3m |  | 23.63 (11.38) [46] | 20.76 (13.36) [49] |  | -2.58 [-7.40, 2.24], .290, 0.21 |
|  | 12m |  | 24.67 (13.19) [48] | 20.89 (11.95) [48] |  | -3.19 [-7.99, 1.61], .191, 0.25 |
| SPS | Pre |  | 32.51 (11.64) [49] | 28.44 (14.34) [50] |  |  |
|  | Mid |  | 19.62 (10.76) [48] | 17.04 (12.49) [48] |  | -1.31 [-4.86, 2.25], .468, 0.11 |
|  | Post |  | 11.98 (9.00) [46] | 8.72 (10.16) [50] |  | -2.01 [-5.56, 1.55], .266, 0.21 |
|  | 3m |  | 10.06 (7.43) [47] | 8.34 (9.97) [49] |  | -0.55 [-4.11, 3.00], .760, 0.06 |
|  | 12m |  | 10.77 (7.52) [48] | 8.44 (9.78) [48] |  | -1.06 [-4.62, 2.49], .555, 0.12 |
| LSAS | Pre |  | 77.69 (17.67) [49] | 78.53 (18.56) [50] |  |  |
|  | Mid |  | 50.35 (19.92) [49] | 47.16 (24.85) [50] |  | -4.08 [-11.54, 3.38], .280, 0.18 |
|  | Post |  | 32.15 (19.60) [48] | 25.82 (18.24) [50] |  | -7.45 [-14.93, 0.03], .051, 0.39 |
|  | 3m |  | 25.89 (16.29) [47] | 23.40 (20.01) [49] |  | -4.47 [-11.99, 3.05], .241, 0.24 |
|  | 12m |  | 33.42 (18.42) [48] | 27.70 (19.51) [48] |  | -7.30 [-14.82, 0.22], .057, 0.38 |
| FNE | Pre |  | 26.37 (3.08) [49] | 25.76 (3.87) [50] |  |  |
|  | Mid |  | 22.29 (6.15) [48] | 20.18 (6.21) [48] |  | -1.60 [-4.70, 1.50], .308, 0.26 |
|  | Post |  | 16.41 (8.37) [46] | 14.57 (7.7) [50] |  | -1.67 [-4.77, 1.43], .287, 0.21 |
|  | 3m |  | 15.36 (8.48) [47] | 13.57 (7.98) [48] |  | -1.79 [-4.89, 1.31], .253, 0.22 |
|  | 12m |  | 15.27 (8.31) [48] | 12.32 (7.97) [48] |  | -2.63 [-5.71, 0.45], .093, 0.32 |
| ADIS | Pre |  | 4.00 (0.93) [49] | 4.07 (1.03) [50] |  |  |
|  | Post |  | 1.94 (1.04) [46] | 1.50 (1.12) [50] |  | -0.49 [-0.90, -0.08], .021, 0.45 |
|  | 3m |  | 1.56 (0.84) [44] | 1.31 (1.14) [46] |  | -0.38 [-0.79, 0.04], .076, 0.37 |
|  | 12m |  | 1.75 (0.94) [44] | 1.49 (1.20) [45] |  | -0.37 [-0.79, 0.05], .081, 0.34 |
| *Components of process composite* | | | | | | |
| SCQ Frequency | Pre |  | 2.96 (0.63) [49] | 2.86 (0.77) [50] |  |  |
|  | Mid |  | 2.02 (0.60) [49] | 1.74 (0.56) [50] |  | -0.26 [-0.46, -0.06], .010, 0.45 |
|  | Post |  | 1.64 (0.51) [48] | 1.42 (0.46) [50] |  | -0.20 [-0.40, -0.01], .044, 0.41 |
|  | 3m |  | 1.43 (0.35) [47] | 1.34 (0.43) [49] |  | -0.09 [-0.29, 0.11], .370, 0.23 |
|  | 12m |  | 1.62 (0.59) [48] | 1.46 (0.50) [48] |  | -0.16 [-0.36, 0.04], .110, 0.29 |
| Measure | Time |  | Unadjusted Mean (SD) [N] | |  | Adjusted difference [95%CI], p value, *d* |
|  |  |  | iCT | CT |  |  |
| SCQ Belief | Pre |  | 48.74 (13.62) [49] | 46.70 (19.36) [50] |  |  |
|  | Mid |  | 26.50 (18.79) [49] | 17.98 (14.24) [50] |  | -8.17 [-13.31, -3.03], .002, 0.49 |
|  | Post |  | 14.96 (13.33) [48] | 8.97 (10.58) [50] |  | -5.86 [-11.02, -0.70], .028, 0.48 |
|  | 3m |  | 10.55 (9.76) [47] | 7.37 (9.60) [49] |  | -3.29 [-8.49, 1.91], .212, 0.34 |
|  | 12m |  | 14.36 (14.50) [47] | 9.53 (12.40) [48] |  | -4.73 [-9.95, 0.49], .075, 0.35 |
| SBQ | Pre |  | 2.44 (0.31) [49] | 2.36 (0.34) [50] |  |  |
|  | Mid |  | 2.00 (0.33) [48] | 1.98 (0.36) [48] |  | 0.02 [-0.13, 0.13], .974, 0.01 |
|  | Post |  | 1.73 (0.30) [46] | 1.69 (0.31) [50] |  | -0.03 [-0.16, 0.10], .647, 0.10 |
|  | 3m |  | 1.64 (0.26) [47] | 1.64 (0.33) [49] |  | <0.001 [-0.13, 0.13], .995, 0.00 |
|  | 12m |  | 1.68 (0.28) [48] | 1.64 (0.37) [48] |  | -0.04 [-0.16, 0.09], .580, 0.11 |
| SAQ | Pre |  | 4.72 (0.55) [49] | 4.69 (0.74) [50] |  |  |
|  | Mid |  | 3.92 (0.84) [48] | 3.80 (0.93) [47] |  | -0.09 [-0.46, 0.27], .612, 0.11 |
|  | Post |  | 3.15 (0.88) [46] | 2.96 (0.88) [50] |  | -0.23 [-0.59, 0.14], .220, 0.26 |
|  | 3m |  | 3.06 (0.91) [47] | 2.85 (1.02) [49] |  | -0.25 [-0.62, 0.11], .175, 0.26 |
|  | 12m |  | 3.12 (1.06) [48] | 2.89 (1.04) [48] |  | -0.24 [-0.61, 0.12], .188, 0.23 |
| Self-Focused Attention | Pre |  | 5.80 (1.02) [49] | 5.52 (1.27) [50] |  |  |
|  | Mid |  | 3.61 (1.58) [49] | 3.36 (1.42) [50] |  | -0.28 [-0.92, 0.36], .389, 0.18 |
|  | Post |  | 3.02 (1.52) [48] | 2.43 (1.34) [50] |  | -0.66 [-1.30, -0.01], .046, 0.45 |
|  | 3m |  | 2.66 (1.48) [47] | 2.46 (1.46) [49] |  | -0.31 [-0.96, 0.34], .349, 0.21 |
|  | 12m |  | 3.05 (1.86) [48] | 3.06 (1.77) [48] |  | -0.05 [-0.70, 0.59], .871, 0.03 |

Note . iCT = Internet-based Cognitive Therapy, CT = Standard (face-to-face) Cognitive Therapy. Table includes everyone who was randomized to iCT or CT, either immediately or at the end of the waitlist. Components of the social anxiety composite: LSAS = Liebowitz Social Anxiety Scale, SPIN = Social Phobia Inventory, SIAS = Social Interaction Anxiety Scale, SPS = Social Phobia Scale, FNE = Fear of Negative Evaluation Scale, ADIS = Anxiety Disorders Interview Schedule Social Anxiety Scale (mean of 0-8 fear and avoidance ratings for 14 social situations). Components of the process composite: SCQ = Social Cognitions Questionnaire, SBQ = Social Behaviours Questionnaire, SAQ = Social Attitudes Questionnaire . Adjusted mean differences based on linear mixed effects models adjusted for baseline scores. *d* is the standardised effect size (Cohen’s d), calculated using the pooled standard deviation.

| Table S5. Additional recovery, remission, responder and improvement criteria used in the social anxiety disorder literature | | | | |
| --- | --- | --- | --- | --- |
| Assessment | iCT | | CT | |
|  | % | (n)^1^ | % | (n)^1^ |
| *Recovery (RCSC)^2^* | | | | |
|  |  |  |  |  |
| Posttreatment | 75% | (36/48) | 80% | (40/50) |
| 3 month Follow-up | 81% | (38/47) | 82% | (40/49) |
| 12 month Follow-up | 71% | (34/48) | 73% | (35/48) |
| *Remission (LSAS <31)^3^* | | | | |
|  |  |  |  |  |
| Pretreatment | 0% | (0/49) | 0% | (0/50) |
| Posttreatment | 50% | (24/48) | 62% | (31/50) |
| 3 month Follow-up | 70% | (33/47) | 69% | (34/49) |
| 12 month Follow-up | 48% | (23/48) | 58% | (28/48) |
| *Responder Status^4^* | | | | |
| Posttreatment | 85% | (41/48) | 96% | (48/50) |
| 3 month Follow-up | 100% | (47/47) | 92% | (45/49) |
| 12 month Follow-up | 85% | (41/48) | 92% | (44/48) |
| *IAPT Reliable Improvement^5^*  Posttreatment  3 month Follow-up  12 month Follow-up | 84%  90%  92% | (41/49)  (44/49)  (45/49) | 92%  96%  92% | (46/50)  (48/50)  (46/50) |
| *Notes:* ^2^ RCSC= reliable and clinically significant change on the Liebowitz Social Anxiety Scale: LSAS (Clark et al, 2006). ^3^LSAS score of 30 or less (Liebowitz et al, 2005). ^4^A reduction of at least 31% on the LSAS (Leichsenring et al, 2013). ^5^ In line with the IAPT Manual (NHS England, 2021) a patient’s score on the SPIN and/or the PHQ-9 drops by the reliable change amount (at least 10 and 6 points respectively) *and* the score on neither increases by the reliable change amount. Patients who did not have a SPIN or PHQ score available at the relevant post-treatment or follow-up assessment are assumed to have not reliably improved. So, the denominators for the % reliable improvement values are n= 49 for iCT and n= 50 for CT, even though a few patients did not provide SPIN (see Table S4) or PHQ-9 (see Table 3) scores at some assessment points. | | | | |
